# Correlation Between IL-10 and microRNA-187 Expression in Epileptic Rat Hippocampus and Patients with Temporal Lobe Epilepsy

**DOI:** 10.3389/fncel.2015.00466

**Published:** 2015-12-02

**Authors:** Walid A. Alsharafi, Bo Xiao, Mutasem M. Abuhamed, Fang-Fang Bi, Zhao-Hui Luo

**Affiliations:** ^1^Department of Neurology, Xiangya Hospital, Central South UniversityChangsha, Hunan, China; ^2^Department of Neurology, Alkhadamat Altebia HospitalAmman, Jordan

**Keywords:** temporal lobe epilepsy, interleukin-10, microRNA-187, anti-inflammation, cytokine

## Abstract

Accumulating evidence is emerging that microRNAs (miRNAs) are key regulators in controlling neuroinflammatory responses that are known to play a potential role in the pathogenesis of temporal lobe epilepsy (TLE). The aim of the present study was to investigate the dynamic expression pattern of interleukin (IL)-10 as an anti-inflammatory cytokine and miR-187 as a post-transcriptional inflammation-related miRNA in the hippocampus of a rat model of status epilepticus (SE) and patients with TLE. We performed a real-time quantitative PCR and western blot on rat hippocampus 2 h, 7 days, 21 days and 60 days following pilocarpine-induced SE, and on hippocampus obtained from TLE patients and normal controls. To detect the relationship between IL-10 and miR-187 on neurons, lipopolysaccharide (LPS) and IL-10-stimulated neurons were performed. Furthermore, we identified the effect of antagonizing miR-187 by its antagomir on IL-10 secretion. Here, we reported that IL-10 secretion and miR-187 expression levels are inversely correlated after SE. In patients with TLE, the expression of IL-10 was also significantly upregulated, whereas miR-187 expression was significantly downregulated. Moreover, miR-187 expression was significantly reduced following IL-10 stimulation in an IL-10–dependent manner. On the other hand, antagonizing miR-187 promoted the production of IL-10 in hippocampal tissues of rat model of SE. Our findings demonstrate a critical role of miR-187 in the physiological regulation of IL-10 anti-inflammatory responses and elucidate the role of neuroinflammation in the pathogenesis of TLE. Therefore, modulation of the IL-10 / miR-187 axis may be a new therapeutic approach for TLE.

## Introduction

Epilepsy is the most prevalent chronic neurological disorder that has been estimated to affect 0.8% of the world’s population. In clinical studies, temporal lobe epilepsy (TLE) is the most common focal forms of drug-resistant epilepsy and characterized by recurrent unprovoked seizures due to neuronal hyperactivity in the brain (Shorvon, 1996). However, biochemical and molecular mechanisms of TLE are still incompletely understood. In the last two decades, extensive studies have provided evidence for the potential role of neuroinflammation in the pathogenesis of human epilepsy (Pitkänen and Sutula, 2002; Vezzani and Granata, 2005).

Interleukin (IL)-10 is a potent antiinflammatory cytokine that has been found to deactivate dendritic cells and macrophages after an inflammatory trigger, which inhibits the pro-inflammatory cytokines production. Proinflammatory cytokines are known to play a critical role in the pathogenesis of TLE (Moore et al., 2001; Vezzani et al., 2002; Li et al., 2011). A number of animal studies and clinical observations suggest an anticonvulsant effect of IL-10 (Kurreeman et al., 2004; Levin and Godukhin, 2007; Ishizaki et al., 2009; Youn et al., 2012). A recent analysis indicated neuroprotective effects of IL-10 against the development of hypoxia-evoked epileptiform activity in rat hippocampal slices (Levin and Godukhin, 2007). In experimental hyperthermic seizures in rat models, the seizure threshold temperature in IL-10 treated rats was significantly upregulated compared to normal controls (Ishizaki et al., 2009). Genetically, the frequencies of the IL-10 592C allele and 1082A/-819C/-592C haplotype, that are reported to be inducers of IL-10, were significantly downregulated in patients with febrile seizures (FS) relative to normal controls, suggesting that IL-10 is genetically associated with FS and confers resistance to FS (Kurreeman et al., 2004; Ishizaki et al., 2009). Furthermore, IL-10 has been found to provide trophic and pro-survival cues to neurons during development and was suggested that exogenously administration of IL-10 may have direct beneficial effects on neurons (Zhou et al., 2009).

miRNAs are a class of short endogenous and small noncoding RNAs, about 18–25 nucleotides in length, that function as key regulators of gene expression at the post-transcriptional level (Kim, 2005). miRNA is partially complementary to one or more mRNA molecules to regulate gene expression of a wide variety of genes involved in critical biological functions via diverse manners such as mRNA cleavage, deadenylation and translational repression. Novel miRNAs is created in non-coding DNA sections from hairpins, duplication and modification of already existed miRNAs or inverted duplications of protein-coding sequences.

Evidence exists that miRNAs are important in the pathogenesis of inflammatory and immune processes (O’Connell et al., 2010). Recently, numerous studies have explored the critical role of miRNAs in TLE using the pilocarpine animal model and patients with TLE, and suggested new therapeutic targets for the treatment of TLE (Kan et al., 2012b; Sano et al., 2012; Alsharafi and Xiao, 2015; Alsharafi et al., 2015). Recent studies have shown that miRNAs are involved in various vital processes, including cell development, cardiac function, cell differentiation, metabolism, apoptosis, cell proliferation, aging, and organ development. Furthermore, miRNAs have been implicated as regulators of about one-third of all protein-coding genes.

miR-187 is a highly neuron-enriched miRNA which has been intensively studied as a cancer-related miRNA and considered as a promising biomarker for the early diagnosis of a wide range of human cancers (He et al., 2012). Recently, miR-187 has been implicated in the regulating of immune and inflammatory processes (Guardia et al., 2013; Suojalehto et al., 2014). Furthermore, miR-187 negatively regulates several proinflammatory cytokines production produced by primary human monocytes (Rossato et al., 2012). Other studies have investigated the relationship between IL-10 and miR-187 in other systems, including monocytes and dendritic cells (Rossato et al., 2012; Guardia et al., 2013).

The aim of the present study was to detect the dynamic expression of IL-10 as anti-inflammatory cytokine and miR-187 as a post-transcriptional inflammation-related miRNA in the hippocampus of rats following pilocarpine-induced status epilepticus (SE) during the three phases of TLE development and in patients with TLE.

## Materials and Methods

### Animals

We started our experiment with fifty eight adult male Sprague– Dawley (SD) rats (6–8 weeks of age, weighing 230–270 g) obtained from the Animal Unit, Central South University, China. Rats were randomly assigned to control C (*n* = 20) and epileptic E (*n* = 38) groups. Animals were kept individually in transparent Plexiglas cages (three animals per cage) on a rotating 12-h light–dark cycle with an ambient temperature of 20–25°C and given *ad libitum* access to food and water. All experimental procedures and animal care were carried out according to protocols approved by the local authorities for animal care, Xiangya Medical College, Central South University.

### Experimental Seizure Model

Thirty eight SD rats were injected with Lithium chloride (125 mg/kg, i.p., Sigma-Aldrich Chemie, Deisenhofen, Germany). Eighteen hours after lithium chloride injections, rats were injected with pilocarpine (20 mg/kg, i.p., Boehringer Mannheim, USA) to induce a generalized SE, followed by a half dose of pilocarpine (10 mg/kg, i.p.) every 30 min if a Racine phase V, tonic clonic, seizure was not elicited (Racine, 1972). The maximum dose for pilocarpine injection was 60 mg/kg. Methylscopolamine (1 mg/kg, i.p.) was administered 15 min before pilocarpine treatment to reduce the peripheral effects of the convulsions and thus enhance survival. SE was terminated after 60 min with injections of chloral hydrate (10%, 3 ml/kg, i.p.). All rats spent approximately 30 min of the 60 min SE phase in a Racine phase V seizure and showed signs of a generalized SE. Following pilocarpine administration, the rats were video-monitored for 2 months (24 h/day), using an infrared ray monitor during phases of early monitoring. Some animals exhibiting seizures were confirmed using electroencephalogram (EEG) recordings that displayed high frequency, high amplitude, poly-spike paroxysmal discharges. Thirty two rats successfully developed SE, four rats died after SE onset and two rats failed to develop SE after repeated injections and were excluded from the study. Saline control rats (*n* = 20) underwent identical treatment but received injections of comparable volume of saline in lieu of pilocarpine and did not exhibit any seizures. The behavior of rats was assessed by an independent observer who was unaware of the treatments given.

Based on epilepsy development phases, each group of rats (experimental and control) were randomly divided into four groups (*n* = 5 for each group): 2 h, 7, 21 and 60 days group. The remaining experimental rats (*n* = 10) were used in the antagomir experiments.

### TLE Patients and Controls

Tissue samples were obtained at surgery from five patients with unilateral drug refractory TLE and typical imaging features and pathological confirmation of hippocampal sclerosis (2 left and 3 right TLE), who had unilateral selective amygdalohippocampectomy. Surgical samples were subjected to routine histopathological examination. As control tissue, five normal hippocampal samples were obtained from patients with no history of any brain diseases. Neuropathologic studies confirmed that the control tissues were normal. Clinical information on patients with TLE and controls was mentioned in our previous work (Table 1; Alsharafi and Xiao, 2015). This study was approved by the Institutional Ethics Committee of Central South University and prior to analysis, written informed consents were obtained from all patients.

**Table 1:** **Clinical information of patients with TLE and controls**.

**ID**	**Sex**	**Age (years)**	**Duration of epilepsy (years)**	**Side of Hippocampus side**	**Antiepileptic drug (AED)**
Epileptic 1	F	10	5	R	PB, LAM, VPA
Epileptic 2	M	29	16	L	VPA, CBZ, TOP
Epileptic 3	M	20	6	R	CBZ, VPA, LAM
Epileptic 4	F	33	24	R	VPA, CBZ, TOP
Epileptic 5	M	24	9	L	CBZ, VPA, LEV
∣rule
**ID**	**Sex**	**Age (years)**	**Post-mortem** **interval (hours)**	**Side of Hippocampus side**	**Causes of death**
∣rule
Control 1	M	65	14	L	Pulmonary carcinoma
Control 2	F	23	19	R	Acute pancreatitis
Control 3	M	19	16	R	Cardiomyopathy
Control 4	M	52	22	L	Respiratory failure
Control 5	F	27	16	L	Hodgkin’s disease

*CBZ, carbamazepine; PB, phenobarbitone; LAM, lamotrigine; LEV, levetiracetam; VPA, valproate acide; TOP, topiramate; R, right; L, left*.

### Rat Tissue Preparation and RNA Isolation

Rats were decapitated at 2 h, 7, 21 and 60 days post-SE or saline administration under deep anesthesia using 10% chloral hydrate (5 ml/kg i.p.). After decapitation, hippocampus was quickly removed from the brain, frozen on dry ice and stored at −80°C until use.

Total RNA isolation was carried out using Trizol method (Invitrogen, Carlsbad, CA, USA) according to the manufacturer’s protocol. RNA quality and quantity were determined at 260/280 nm using a NanoDrop ND-1000 spectrophotometer (NanoDrop Technologies, Thermo Scientific, USA).

### IL-10 Expression by qPCR in the Hippocampi of Rats and TLE Patients

Total hippocampal RNA (1 μg) was reverse-transcribed using a RevertAid First Strand complementary DNA (cDNA) Synthesis Kit (Thermo Scientific’s Fermentas Molecular Biology Tools). PCR was performed in a total volume of 20 μL using the Platinum SYBR Green qPCR Super Mix UDG (Invitrogen, USA) and ABI Mx3000P QPCR System (Stratagene) according to the manufacturer’s instructions.

The relative expression level for IL-10 was calculated by the comparative CT method. β-actin was used as an internal control.

### miR-187 Expression by qPCR in the Hippocampi of Rats, and TLE Patients

cDNA synthesis was generated using the NCode^TM^ VILO^TM^ miR cDNA Synthesis Kit (Invitrogen, USA). qPCR reaction was performed in a total volume of 20 μL using the EXPRESS SYBRR GreenER^TM^ qPCR SuperMix Universal kit (Invitrogen, USA) which contains EXPRESS SYBR^°ledR^ GreenER^TM^ qPCR Supermix, Universal qPCR Primer, and Rox Reference Dye according to the manufacturer’s instructions. The expression of the U6 small nuclear RNA gene was used as an internal control. The relative expression level for miR-187 was calculated using the comparative CT method.

### Western-Blot Analysis

The cytoplasmic extracts (30 μg total protein) were separated by 12% sodium dodecyl sulfate–polyacrylamide (SDS-polyacrylamide) gel and transferred onto a polyvinylidene fluoride (PVDF) membrane using the Bio-Rad system (Bio-Rad, USA) before blocking with TBST containing 5% nonfat-milk for 2 h at room temperature with gentle shaking. Three washing steps of 10 min each were performed after blocking and incubation with the antibodies. The membrane was incubated overnight at 4°C with polyclonal antibodies against IL-10 (Abcam, Hong kong) at 1:500 dilutions and subsequently incubated with a horseradish peroxidase conjugated sheep anti-rabbit IgG (Amersham Biosciences, Piscataway, NJ, USA) at 1:3000 dilutions at 37°C for 1 h. β-actin expression was used as an internal control. The reactions were detected using enhanced chemiluminescence reagents (ECL; KPL, USA). Integrated optical density (IOD) values of IL-10 protein and β-actin were measured. The relative expression amount of IL-10 protein was determined by the ratio of IL-10 protein/β-actin.

### Culture of Hippocampal Neurons

Primary hippocampal neurons were prepared from embryonic day 18 (E18) rats as previously described (Brewer et al., 1993). Rats were decapitated and their brains immediately fetched and placed in phosphate buffered saline (PBS) at 4°C. After removing blood vessels and pia mater, the hippocampi were aseptically dissected. Tissues were minced, washed, centrifuged, dissociated with dissociated with papain, triturated, and plated on coverslips (25 mm) coated with poly-L-lysine (Becton Dickinson) in in a medium consisting of 85% high-glucose Dulbecco’s Minimum Essential Medium (Invitrogen, USA), 15% fetal bovine serum (Invitrogen, USA), and penicillin–streptomycin (106 U/l). Three hours later, the plating medium was changed to feeding medium [neurobasal medium (98%; Invitrogen, USA) supplemented with B27 (2%; Invitrogen, USA), 2 mM glutamine, and penicillin–streptomycin (106 U/l)] and cultured at 37°C under 95% atmosphere and 5% CO2. Half of the feeding medium was changed with fresh one every 3 days. All experiments were performed on the 14th–15th day-in-vitro (DIV) to allow adequate neuronal maturation.

Neurons were randomly divided into four groups: a control group, a LPS-stimulated group, LPS + IL-10 stimulated group and LPS + anti-IL-10 Ab group. In the control group, neurons were cultured without serum Neurobasal-B-27 medium for 30 h to explore the expression of miR-187 on the resting neurons. While in the stimulated group, neurons were cultured without serum Neurobasal-B-27 medium for 6 h and then with Neurobasal-B-27 medium and one or more of the following reagents alone or in combination: recombinant rat IL-10 200 U/mL (abnova), anti-rat IL-10 monoclonal antibody 1 μg/mL (Clone 2G101H7; Biocompare), and LPS 100 ng/mL derived from *Escherichia coli* (Sigma) for 24 h to explore the effect of IL-10 stimulation on miR-187 expression.

### miR-187 Antagomir Experiments

Briefly, miR-187 expression in rat hippocampus following SE was antagonized using an antagomir that specifically and efficiently targets miR-187. A miR-187 antagomir or an antagomir-control (Ribo-Bio Co., Ltd., China) was dissolved in an artificial CSF at a concentration of 20 nmol/mL (1 nmol/50μl for each rat) and infused at a very slow rate by micro-syringe into the lateral ventricle of the epileptic rat (*n* = 10) as previously described (Löscher, 2002). The experiment began at 60 min and ended at 4 h after SE onset. At 7 days following SE onset, rats were decapitated under deep anesthesia using chloral hydrate (10%, 5 ml/kg, i.p.) and hippocampal tissue was quickly removed for detection of miR-187 expression and detection of its critical downstream molecules IL-10.

### Statistics

All of the data are expressed as means ± standard deviation (SD). Statistical significance was evaluated using Student’s *t*-test, or one-way ANOVA performed using the SPSS 13.0 software. The value of *p* < 0.05 was considered to be statistically significant.

## Results

### Behavior and Seizures in a Rat Model

In our experiment, pilocarpine was injected in a dose of 20 mg/kg, i.p. in pretreated rats with lithium chloride to evoke SE in a rat model. SE was terminated by chloral hydrate (3 ml/kg, i.p.) after 60 min. Following Lithium chloride treatment, no behavioral changes were observed in the treated animals, whereas significant behavioral changes were markedly observed following a 5–35 min injection of pilocarpine with akinesia, ataxic lurching, tremor, head bobbing, masticatory automatisms with myoclonus of facial muscles, and wet dog shakes at onset. In each pilocarpine-treated animal, clinical signs of seizure activity were observed. All rats exhibited a well-defined pattern of behavior following pilocarpine injections. Behavioral changes were consistent with the features of human TLE. Thirty two rats successfully developed SE, four rats died after SE onset and two rats failed to develop Racine stage IV-V of SE after repeated injections and were excluded from the study. Saline control rats (*n* = 20), which received injections of comparable volume of saline in lieu of pilocarpine, did not exhibit any seizures. Following pilocarpine administration, the rats were video-monitored for 2 months (24 h/day), using an infrared ray monitor during phases of early monitoring. Two experimental rats were died between 24–72 h following SE as a result of complications of seizures. The remaining animals control group (*n* = 20) and experimental group (*n* = 30) were kept individually in transparent Plexiglas cages (three animals per cage) to be used for further experiments. The acute seizure and acute control groups were sacrificed 2 h after SE. The latent seizure and latent control groups were sacrificed 7 days and 21 days post-SE without any behavioral changes or seizures. Spontaneous seizures occurred mainly 60 days post-SE. Seizures were generally characterized by a focal onset (immobility, mechanical mutation, mouth clonus, forelimb clonus), occasionally culminating in a generalized convulsive stage, lasting about 30 s to 1.5 min. Chronic seizures occurred 60 days post-SE. At this time point, all of the remaining pilocarpine-induced rats manifested with chronic TLE, experiencing seizures one to several times per day with symptoms as described above. Some animals exhibiting seizures were confirmed using EEG recordings that displayed high frequency, high amplitude, poly-spike paroxysmal discharges.

### Dynamic Expression Pattern of IL-10 in Rats During the Three Phases of TLE Development

**Figure 1:**
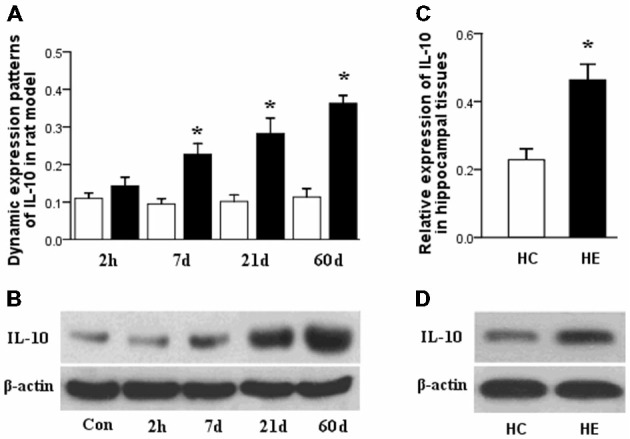
**(A)** Interleukin (IL)-10 dynamic expression patterns in the latent and chronic phases of TLE development in a rat model. qPCR results showed significant upregulation occurred in these two phases of TLE, with higher expression in the chronic phase. **(B)** IL-10 protein expression by western blot in the three phases of TLE development in a rat model also showed similar dynamic changes as gene expression. **(C)** IL-10 expression in hippocampal tissues obtained from patients with TLE and normal controls. qPCR results showed significant upregulation occurred in the TLE patients compared to normal controls. **(D)** IL-10 protein expression by western blot also showed upregulation in the patients’ tissues compared to normal controls. (Data are presented as the mean ± sprague–dawley (SD), ^*^p < 0.05; *n* = 5/group). HC, human control; HE, human epileptic.

qPCR results showed significant upregulation of IL-10 gene expression in the rat hippocampal tissues during the latent and chronic phases of TLE development (^*^p < 0.05) with higher expression at 21 days compared to 7 days and showed its highest expression in the chronic phase (60 days post-SE). In the acute phase (2 h post-SE), IL-10 expression levels were no different between the epileptic and control groups. In rat hippocampal tissues, IL-10 expression was normalized to that of β-actin (Figure 1A). Our results were confirmed by detecting IL-10 protein expression by western blot, which showed similar dynamic changes as gene expression (Figure 1B).

### Relative Expressions of IL-10 in Patients with TLE

qPCR results showed also significantly upregulation of IL-10 gene expression in the hippocampal tissues obtained from patients with TLE compared to tissues from normal controls (^*^p < 0.05). We confirmed our results by detecting the expression of IL-10 protein by western blot, which also showed upregulation in the patients’ tissues compared to normal controls. In the patients’ tissues, IL-10 expression was normalized to that of β-actin (Figures 1C,D).

### miR-187 Expression in Rats During the Three Phases of TLE Development

**Figure 2:**
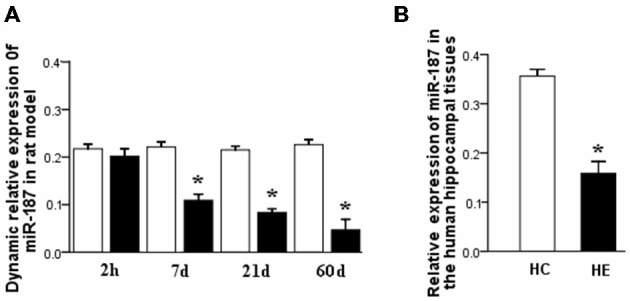
**(A)** miR-187 relative dynamic expression in the three phases of TLE development in a rat model. qPCR results showed significant downregulation occurred in the latent and chronic phases compared to controls, with lowest expression in the chronic phase. In the acute phase, no significant deregulation was observed. **(B)** Relative expression of miR-187 in the hippocampal tissues obtained from patients with TLE and normal controls. qPCR results showed significant downregulation occurred in the TLE patients compared to normal controls (*n* = 5/group; ^*^p < 0.05). HC, human control; HE, human epilepsy.

qPCR results showed significant downregulation of miR-187 expression in the rat hippocampal tissues in the latent and chronic phases of TLE development (^*^p < 0.05). Expression was lower in the 21 days group compared to the 7 days group and showed its lowest expression in the chronic phase (60 days post-SE). Notably, no significant deregulation was detected in the acute phase (2 h post-SE). In rat tissues, miR-187 expression was normalized to that of the U6B small nuclear RNA gene (rnu6b; Figure 2A).

### miR-187 Expression in Patients with TLE

qPCR results showed also significant downregulation of miR-187 expression in the hippocampal tissue of TLE patients relative to controls (^*^p < 0.05). In the tissues obtained from patients, miR-187 expression was normalized to rnu6b (Figure 2B).

### Effect of Modulation of IL-10 on miR-187 Expression in the Neurons

**Figure 3:**
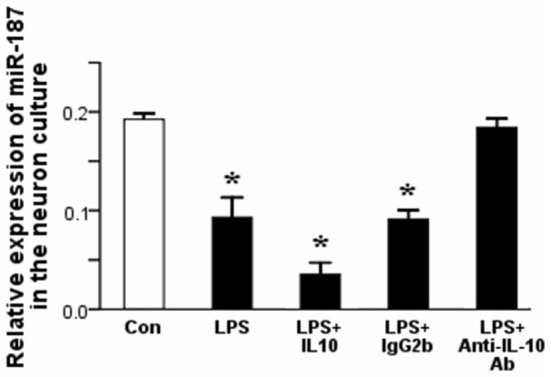
**qPCR results showed a significant downregulation of miR-187 in the neurons after treatment with LPS than control neurons**. A dramatically downregulation of miR-187 in the neurons was detected after treatment with LPS + IL-10 than control neurons, while simultaneous addition of anti-IL-10 Ab to LPS-stimulated neurons restored the expression of miR-187 (Data are presented as the mean ± SD, ^*^p < 0.05; *n* = 5/group). LPS, lipopolysaccharides; Ab, antibody.

qPCR results revealed a significant downregulation in miR-187 expression in the neurons after stimulation with LPS than controls (^*^p < 0.05). Furthermore, simultaneous addition of IL-10 to LPS-stimulated neurons suppressed the expression of miR-187 compared to LPS-stimulated neuron group, whereas simultaneous addition of anti-IL-10 Ab to LPS-stimulated neurons restored the expression of miR-187 (Figure 3). These data point to miR-187 as a LPS-related miRNA up regulated by IL-10. miR-187 expression was normalized to that of the rnu6b.

### Effect of miR-187 Antagomir in IL-10 Expression Level

**Figure 4:**
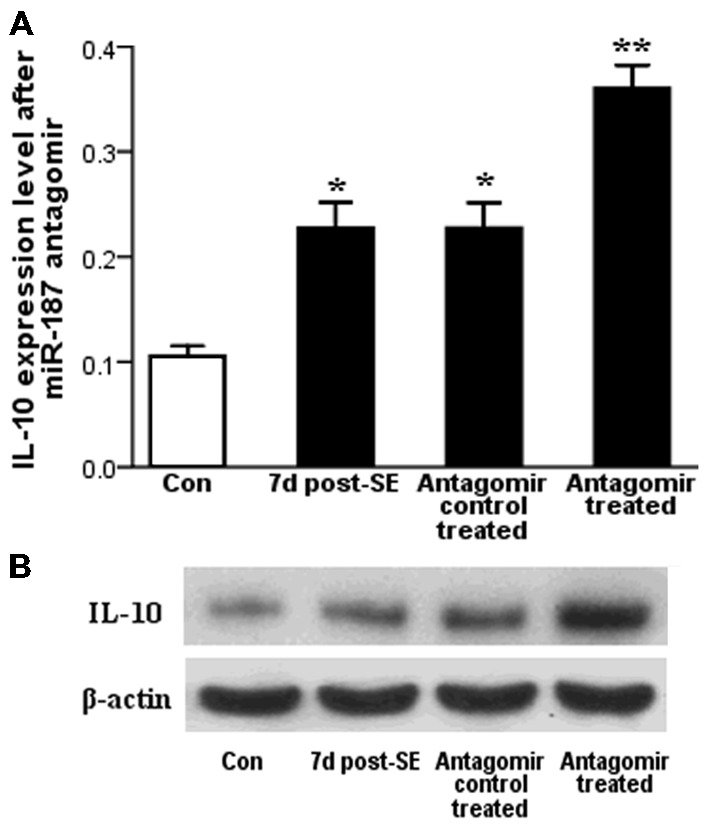
**The results of the miR-187 antagomir experiment. (A)** qPCR results showed significant upregulation of IL-10 expression at 7 days post-status epilepticus (SE) in the rat hippocampal both before and after miR-187 antagomir/antagomir-control treatments. **(B)** Detection of IL-10 protein expression by western blot showed similar dynamic changes as gene expression (^*^p < 0.05, ^**^p < 0.01, *n* = 5/group).

qPCR results showed that IL-10 expression level is increased at 7 days post-SE in the rat hippocampal tissues both before and after miR-187 antagomir/antagomir-control treatments. Stimulation of IL-10 expression level was markedly observed in the miR-187 antagomir group compared to the antagomir-control-treated group (Figure 4A). We confirmed our results by western blot that also detected an upregulation in the expression of IL-10 protein at 7 days post-SE in the rat hippocampus when compared with control. The expression of IL-10 protein at 7 days post-SE in the rat hippocampus increased in the miR-187 antagomir-treated group when compared with the antagomir-control-treated group (Figure 4B).

## Discussion

Despite the available extensive literature of studies on epilepsy, no treatments to avoid or reverse the development of epilepsy have been developed. Currently available antiepileptic treatments are not effective in 1/3 of patients. Recently, lines of research are beginning to investigate the role of inflammation and cytokine release in seizures and epileptogenesis in experimental models and human TLE (Vezzani et al., 2002; Dubé et al., 2010; Youn et al., 2012). Auvin et al. (2010) found that inflammation worsens the consequences of epilepsy in the animal models. Since IL-10 plays a critical role in immune and inflammatory responses and a defect in its production has been attributed to certain inflammatory diseases. Better understanding of which molecules and functional mechanisms those regulate the expression of this anti-inflammatory cytokine may offer new therapeutic targets for the treatment of TLE. In recent years, altered miRNA expression in brain has been extensively studied for its critical function related to a wide range of neurological diseases including epilepsy. Several target studies, as well few genome-wide miRNA expression profiling studies, have reported abnormal miRNA expression in tissue with mesial temporal sclerosis, both in patients and in animal models. Interestingly, these studies revealed a tight correlation between miRNA deregulations and neuroinflammation, seizure-induced neuronal death and other related biological pathways. In our previous studies we demonstrated the expression profile of miRNAs in the hippocampus in a rat model of TLE and showed the neuroprotective effect of targeting miR-34a (Hu et al., 2011, 2012). Importantly, inflammation, stress signaling and neuronal excitation are among the pathways most impacted. Overall, it is now clear that miRNAs regulate a wide variety of pathogenic and functional mechanisms during TLE development.

In the present study, we first demonstrated an association between IL-10 as an anti-inflammatory cytokine and miR-187 as a post-transcriptional inflammation- related miRNA during the three phases of TLE development in a rat model, and we confirmed our results by detecting their expressions in patients with TLE, which are equal to the chronic phase in the rat model. In addition, we examined the effect of modulation of IL-10 expressions on miR-187 expressions* in vitro* on the neuron level and the effect of antagonizing miR-187 on the expression of IL-10 to identify the correlation between miR-187 and IL-10 expression levels. We observed that IL-10 secretion and miR-187 expression levels are inversely correlated after SE, in which IL-10 expression was highest in the chronic phase, when expression of miR-187 was at its lowest level; miR-187 expression was highest in the latent phase, when IL-10 expression was at its lowest level. In the acute phase no significant deregulation was detected in both IL-10 and miR-187. This suggests their underlying functions in the process of TLE development.

In the acute phase, IL-10 expression started to be detectable after 2 h, however, no statistical significance was found. In association with our observation, Youn et al. (2012) also found that IL-10 expression takes from 2–3 days to be significantly detectable in plasma in neonatal seizures induced by hypoxic-ischemic encephalopathy.

In the latent phase, IL-10 showed a significant upregulation with a higher expression at 21 days post-SE compared with 7 days group. The rapid and prominent expression of IL-10 in the latent phase may also corresponds to the changes in gene expression involved in inflammatory response occur during latent phase (Aronica et al., 2000, 2001; Gorter et al., 2006).

In the chronic phase, IL-10 showed its highest expression. These results corroborate other findings that showed an increased in the IL-10 production of chronic epileptic rats (Costa-Ferro et al., 2011; Ferrazoli et al., 2013). Consistent with the chronic phase findings in rat, we revealed an upregulation of IL-10 in patients with TLE, which has previously been shown in TLE patients (Kan et al., 2012a). This upregulation of IL-10 expression was associated with the onset of chronic spontaneous seizures. Our results support the role of chronic inflammation as an insistent guest in the brain undergoing epileptogenesis, being manifested by activations of multiple inflammatory markers including cytokines. On the other hand, our findings support the idea that IL-10 promotes an anti-inflammatory action, mediating inflammatory cascades triggered by induced SE. Therefore, upregulation of IL-10 during epileptogenesis and chronic phase of TLE development could be crucial for arresting the development of TLE.

Interestingly, miR-187 exhibited an opposite expression manner to IL-10 which was significantly downregulated during the latent and chronic phases of TLE development in a gradual manner. In the acute phase, miR-187 expression was almost equal to expression values in the control group. Notably, several profiling studies have shown that miR-187 is not deregulated in the acute phase of TLE (Hu et al., 2011, 2012), which coincides with our findings in this study.

The latent phase statistically reduced the expression of miR-187 compared to normal controls and its expression being more decreased in 21 days than in 7 days group. This may relate to the change in IL-10 expression which exhibits an opposite dynamic pattern. The same expression was also found at 7 days post-SE by Bot et al. (2013). In their study, they also evaluated the prolonged expression of miR-187 in tissues collected from different time points (7–90 days post-SE) which was also downregulated. This significant decrease in miR-187 expression which is associated with the significant increased level of IL-10 expression in this animal model support the hypothesis that miR-187 may represent an attempt to regulate the anti-inflammatory response of IL-10 by inducing its expression level (Rossato et al., 2012).

In the chronic phase, miR-187 expression reached its lowest, which was confirmed by its downregulation in patients with TLE. In association with our observation, several profiling studies have shown that miR-187 is downregulated in the chronic phase of TLE either in experimental models or patients with TLE. Kretschmann et al. (2015) found a significant downregulation of miR-187 expression at 28 days pilocarpine-induced SE in mice. Bot et al. (2013) also reported a downregulation in this miRNA at 30 days after SE induced by amagdala electrical stimulation. Another study by Gorter et al. (2014) also detected a significant decreased of miR-187 at 3–4 months following SE induced by electrical stimulation of angular bundle in rats. McKiernan et al. (2012) found that mature miR-187 was decreased in association with dicer loss in patients with TLE and HS. This suggests that miR-187 may play a special role in the pathogenesis of TLE. Several studies have suggested a link between miR-187 and chronic human inflammatory diseases (Holliday et al., 2011; Guardia et al., 2013; Suojalehto et al., 2014). In previous animal studies and clinical observations, inflammation-related miRNAs were also detected deregulated during the chronic phase of TLE (Omran et al., 2012; Ashhab et al., 2013). Therefore, the dramatic downregulation of miR-187 expressions in both rat models and patients with TLE during epileptogenesis and chronic phase indicates their underlying functions in the process of TLE development.

Recently, many reports have provided insights into the involvement of miRNAs in the posttranscriptional regulation of cytokine genes and vise versa (Liu et al., 2011; Omran et al., 2012; Ashhab et al., 2013).To investigate the relationship between IL-10 and miR-187, we evaluated their relationship on neurons. Furthermore, we explored the effect of antagonizing of miR-187 in IL-10 expression levels. IL-10 alone has been shown to be a relatively weak stimulus, but it strongly synergized with LPS in alteration of miR-187 expression (Rossato et al., 2012). Several lines of evidence clearly point to the requirement for IL-10 in the reduction of miR-187 expression in LPS-activated neurons. Stimulation of the neurons with LPS leads to a significant downregulation of miR-187 expression compared to the resting state. miR-187 expression failed to be downregulated when IL-10 is blocked with anti–IL-10 antibody. Furthermore, IL-10 directly abolished LPS-reduced miR-187 transcription, which is potentiated in the presence of IL-10–blocking antibodies. Therefore, downregulation of miR-187 in response to LPS may be required for proper IL-10 production during TLE. On the other hand, antagonizing of miR-187 function by antagomir in the post-SE rat hippocampus significantly increased the expression levels of IL-10 after antagonizing miR-187. Thus, miR-187 mediated posttranscriptional control could be involved in fine tuning the requirement level of IL-10 expression in anti-inflammatory responses following SE.

Overall, our observations represent evidence that miR-187 are strongly involved in the anti-inflammatory functions of IL-10 during TLE. These results are supported by findings that suggest a major role for miR-187 in the physiological regulation of IL-10–driven anti-inflammatory responses by inhibiting several proinflammatory cytokins production including TNF-α, IL-6 and IL-12p40 (Rossato et al., 2012). However, further studies are needed to evaluate the exact underlying mechanisms.

## Conclusion

The current study shows that IL-10 and miR-187 are mediated in the pathogenesis of TLE development and their opposite expression patterns in the phases of TLE development may suggest an interactive relationship between the two markers, suggesting that miR-187 may play a critical role in the modulation of the neuroinflammatory response during the pathogenesis of TLE. Moreover, our findings support the idea that the administration of IL-10 may be helpful in preventing TLE development. Therefore, modulation of the IL-10/miR-187 axis may be a novel therapeutic target for TLE.

## Conflict of Interest Statement

The authors declare that the research was conducted in the absence of any commercial or financial relationships that could be construed as a potential conflict of interest.
